# Recalcitrant lymphocytoma cutis successfully treated with mycophenolate mofetil

**DOI:** 10.1177/2050313X211025104

**Published:** 2021-06-14

**Authors:** Farhan Mahmood, Iris YH Teo, Mark G Kirchhof

**Affiliations:** 1 Faculty of Medicine, University of Ottawa, Ottawa, ON, Canada; 2 Department of Pathology and Laboratory Medicine, University of Ottawa, Ottawa, ON, Canada; 3 Division of Dermatology, Department of Medicine, University of Ottawa and The Ottawa Hospital, Ottawa, ON, Canada

**Keywords:** Lymphocytoma cutis, cutaneous lymphoid hyperplasia, mycophenolate mofetil, immunosuppressant

## Abstract

Lymphocytoma cutis reflects an exaggerated local immunologic reaction to a stimulus presenting on the head, neck, or upper extremities as a firm 1–3 cm erythematous and/or violaceous plaque or nodule. However, lymphocytoma cutis may be difficult to treat due to the variety of causative agents and the lack of reported successful treatments and outcomes. Here, we present a case of 68-year-old female with recalcitrant lymphocytoma cutis resistant to other first-line therapies including tacrolimus ointment and steroids. The red plaque on the patient’s left cheek was eventually treated with mycophenolate mofetil. Mycophenolate mofetil was an accessible and effective therapeutic option to treat lymphocytoma cutis with minimal side effects.

## Introduction

Lymphocytoma cutis (LC), also known as cutaneous lymphoid hyperplasia, reflects an exaggerated local immunologic reaction to a stimulus.^1^ Possible stimuli include arthropod bites, tattoos, metal implants, contact allergens, vaccinations, and medications, herpes zoster, and Lyme borreliosis.^2^ Clinically, LC presents on the head, neck, or upper extremities as a firm 1–3 cm erythematous and/or violaceous plaque or nodule. Nodules can vary from amalgamated papules to larger nodules resembling panniculitis.^3^ Although treatment of LC is often conservative, LC may be difficult to treat due to the variety of causative agents and the lack of reported successful treatments and outcomes.^4^ Conventional treatments for LC are dependent on the etiology of the condition. Common treatment options include topical or intralesional steroids, antibiotics (i.e. amoxicillin), or laser;^4^ mycophenolate mofetil (MMF) is not conventionally used. Here, we present a case of recalcitrant LC treated with MMF. This therapy is an accessible and effective option to treat LC with minimal side effects.

## Case report

**Figure 1.**
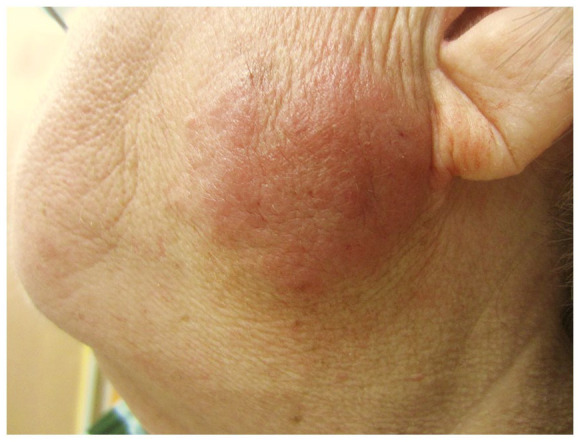
Lymphocytoma cutis—firm, pink to red plaque on the left cheek, 5 × 6 cm in size.

A 68-year-old woman presented with a pink to red plaque on the left cheek, 5 × 6 cm in size (Figure 1). Patient described it as itchy. The plaque was firm to palpation, and there was no lymphadenopathy on exam. Patient’s past medical history included hypertension, coronary stenting, fibromyalgia, osteoarthritis, and gastroesophageal reflux disease (GERD).

A skin biopsy of the left cheek showed a very dense nodular lymphocytic infiltrate with germinal centers containing tangible body macrophages (Figure 3). Germinal centers were surrounded by a small rim of mantle lymphocytes admixed with eosinophils. The epidermis was normal with a grenz zone. All markers were benign including a normal kappa/lamda ratio, and the BCL6 germinal centers were BCL2 negative. Extension to the fat was observed with no obvious lobular panniculitis. Upon review by five pathologists, the patient was diagnosed with LC. A computerized tomography scan of the body and head/neck was negative of other pathologies.

**Figure 2.**
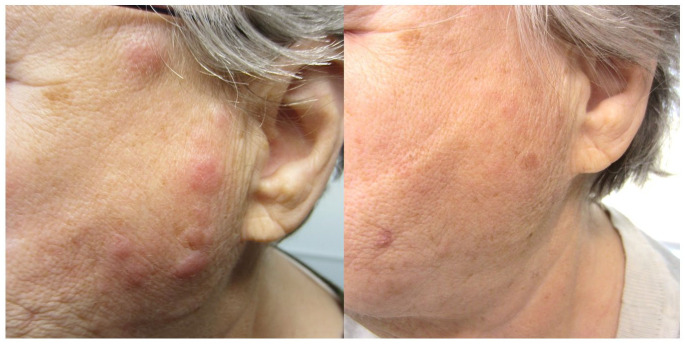
Lymphocytoma cutis treated with mycophenolate mofetil after 3 months (left) and 6 months (right).

Patient was initially treated with tacrolimus 0.1% ointment and prednisone 40 mg PO OD for 1 month, which helped with the pruritus, but did not resolve the lesion. Tacrolimus 0.1% ointment was continued. Intralesional steroid injections (triamcinolone acetonide 10 mg/mL with ~0.2 cc, injected every 0.5 cm—total of 15 mg) every 4–6 weeks for 6 months were also trialed with only 30%–40% improvement. Patient was then prescribed hydroxychloroquine 400 mg PO OD for 3 months, followed by methotrexate 15 mg PO once a week for 3 months, both being ineffective. Cyclosporine 50 up to 150 mg PO BID was given over 6 months with 50%–60% improvement but was discontinued due to worsening hypertension. MMF 500 mg BID for 3 months was trialed resulting in 50% improvement with no side effects. The MMF dose was increased to 1000 mg BID for 3 months with complete clearance (Figure 2). However, the patient developed mild anemia. Thus, the MMF dose was titrated to optimize response and limit side effects. The dose varied between 500 and 1000 mg in the morning and 250–500 mg in the evening based on the clinical response and blood work results. Informed consent was obtained from the patient to discuss their case and utilize their pictures.

**Figure 3.**
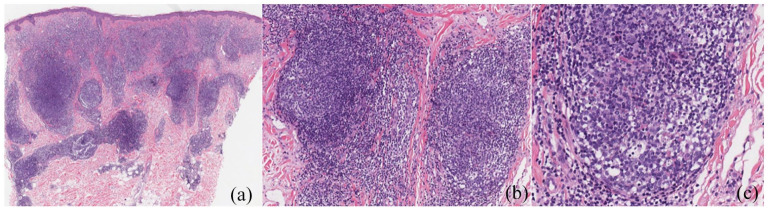
(a) 2× magnification, dense nodular/follicular architecture extending into the mid/deep dermis, and the grenz zone; (b) 10× magnification, polarization of the follicles; (c) 20× magnification eosinophils in the bottom half (difficult to appreciate), tingible body macrophages.

## Discussion

LC can be characterized based on the presence of variable numbers of medium- to large-sized atypical and ordinary lymphocytes and other inflammatory cells. Most cases consist of both B- and T-cells with macrophages and dendritic cells. B-cell predominant LC, as in our case, presents as superficial and deep infiltrates of lymphocytes with histiocytes, plasma cells, and eosinophils, whereas T-cell predominant LC consists of CD4+ T helper lymphocytes with CD8+ cytotoxic T-cells.^2,3,4^

Although LC lesions can resolve spontaneously once etiologic agents are removed, the lack of standardized treatment guidelines, vast amounts of causative agents, and the lack of reported effective treatment options still make LC difficult to treat. First-line therapies include surgery, topical or intralesional corticosteroids, laser, drug discontinuation (in drug-induced LC), antiretroviral therapy, antibiotics, topical tacrolimus, radiotherapy, thalidomide, and sun-protection. Second-line treatments include psoralen plus ultraviolet A, long-wave ultraviolet A, 5-aminolevulinic acid photodynamic therapy, topical imiquimod and tacrolimus, and hydroxychloroquine.^4^ Unconventionally, intravenous rituximab has also been shown to treat recalcitrant pseudolymphoma previously unresponsive to corticosteroids and laser therapy.^5^

In our case, recalcitrant LC was treated with an unconventional therapy, MMF—an immunosuppressive prodrug of mycophenolic acid (MPA). MPA is an inhibitor of the type II isoform of IMDPH. IMDPH is a rate-limiting enzyme in the de novo synthesis of guanosine nucleotides, which are required for T- and B-cell lymphocyte proliferation.^6^ MPA also inhibits leukocyte recruitment, glycosylation of lymphocytic glycoproteins, and endothelial prostaglandin E_2_ production.^6,7^ This results in a pronounced cytostatic effect on lymphocytes. Thus, the efficacy of MMF in LC may be associated with the lack of B- and T-cell lymphocyte proliferation via the inhibition of guanosine nucleotide synthesis.

The steroid-sparing effects and relative lack of toxicity are some of the benefits of using MMF. MMF is an efficacious therapeutic option for patients unable to tolerate other medications due to unbearable side effects, such as hypertension from cyclosporine in our case, and to treat severe, refractory inflammatory skin diseases as either monotherapy or adjuvant therapy. MMF is effective in treating inflammatory dermatologic conditions including psoriasis, autoimmune blistering disorders, dermatitides, and connective tissue disorders.^8^

Common side effects of MMF include gastrointestinal effects, which are dose-dependent, including diarrhea, nausea, vomiting, abdominal pain, anal tenderness, soft stools, frequent stools, and constipation.^8^ Hematologic side effects include anemia, which occurred in our case, leucopenia, and thrombocytopenia.^8^ Genitourinary symptoms are more common in the first year of therapy, which include dysuria, urgency, and frequency. High incidences of opportunistic infections have also been reported, particularly in MMF doses of 2 g daily.^8,9^ MMF has also been shown to increase the risk of lymphoma and other malignancies^10^.

MMF is contraindicated in patients with hypersensitivity to MMF, MPA, or other acidic compounds of MMF and patients allergic to polysorbate 80. MMF is not recommended in pregnant women due to its teratogenicity.^10^

To our knowledge, this is the first published report of recalcitrant LC successfully treated with MMF. MMF should be considered as an effective therapy for recalcitrant LC.
